# Correction: Wang, F., et al. Effect of Low-Frequency Electromagnetic Casting on Micro-Structure and Macro-Segregation of 5A90 Alloy Ingots. *Materials* 2020, *13*, 2720

**DOI:** 10.3390/ma14030572

**Published:** 2021-01-26

**Authors:** Fuyue Wang, Xiangjie Wang, Jianzhong Cui

**Affiliations:** 1 Key Lab of Electromagnetic Processing of Materials, Ministry of Education, Northeastern University, Shenyang 110819, China; jzcui@mail.neu.edu.cn; 2 College of Materials Science and Engineering, Northeastern University, 314 Mailbox, Shenyang 110819, China

In the original article [1], there was a mistake in Figure 4 as published. The scale of vertical axis is wrong. The corrected Figure 4 appears below.

There was also an error in the original article. A correction has been made to Section 3.2. Macro-Segregation, paragraph 1. The sentence “There is the large segregation rate of Mg, which appeared close to the surface of the ingot, with a maximal relative segregation rate of 1.20%” should be replaced with “There is the large segregation rate of Mg, which appeared close to the surface of the ingot, with a maximal relative segregation rate of 12.0%”.

The corrected paragraph is present below:

The concentration of alloying elements at different positions of the 5A90 alloy ingots was detected using ICP-AES, and the degree of segregation can be expressed as the relative segregation rate as shown in Equation (1).

(1) $$\Delta C=\frac{c_{i}-c_{o}}{c_{o}}$$

where *c_i_* is the concentration of alloying elements detected at each position, and *c_o_* is the initial concentration of alloying elements. Figure 4 shows the relative segregation rate of the major alloying elements (Mg and Li) versus different positions of the 5A90 ingots. It could be seen from the value of the relative segregation rate that the Mg exhibited a higher segregation tendency as compared to Li. This is due to the different equilibrium distribution coefficients of Mg and Li in aluminum (k_Mg_ < k_Li_). There is the large segregation rate of Mg, which appeared close to the surface of the ingot, with a maximal relative segregation rate of 12.0%. The ingots prepared by DCC and LFEC had a nearly identical segregation pattern. Negative segregation occurred in the center and strong positive segregation occurred on the ingot’s surface, accompanied by a strong negative segregation zone close to the surface. However, the degree of segregation was obviously alleviated by LFEC through the whole cross-section, from surface to center. Depending on the results above, LFEC can effectively improve alloying element macro-segregation of the 5A90 alloy ingot and narrow down the strong negative segregation zone which occurs near the surface of the ingot.

The authors apologize for any inconvenience caused and state that the scientific conclusions are unaffected.

## Figures and Tables

**Figure 4:**
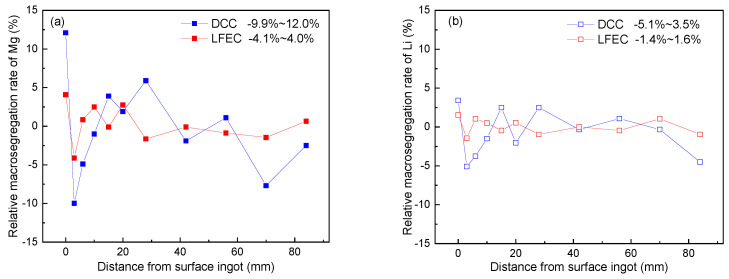
Relative segregation rate of alloying element of 5A90 ingots cast by DCC and LFEC with 10 Hz/100 A. (**a**) Mg; (**b**) Li.
